# Identifying Culturally Relevant School Support Profiles and Links to Academic Functioning in Adolescents

**DOI:** 10.1007/s10964-024-02098-0

**Published:** 2024-10-09

**Authors:** Maciel M. Hernández, Olga Kornienko, Jennifer M. Figueroa, Marita Coker, Karenina Paredes, Caroline Toth, Julia P. Carrillo, Adam A. Rogers, Thao Ha

**Affiliations:** 1 Department of Human Ecology, University of California, Davis, CA USA; 2 Department of Psychology, George Mason University, Fairfax, VA USA; 3 Department of Psychology, Arizona State University, Tempe, AZ USA; 4 School of Family Life, Brigham Young University, Provo, UT USA

**Keywords:** Academic functioning, Adolescence, Culturally relevant school support, Teacher support

## Abstract

There is limited knowledge about patterns of adolescents’ experiences of general teacher support and support for critical consciousness and cultural competence development in school settings, which are key experiences proposed to promote academic functioning. Furthermore, less is known about potential developmental and ethnic-racial differences in these patterns. Using a person-centered approach, this study examined culturally relevant school support profiles in a sample of sixth and ninth grade students (*N* = 717; 49.9% girls) from the U.S. Southwest. Participants were aged 10 to 18 years (*M* = 13.73; *SD* = 1.54) and were ethnoracially diverse (31.8% Hispanic/Latinx, 31.5% Multiethnic, 25.7% White, 7.3% Black or African American, 1.4% Asian American or Pacific Islander, 1.4% American Indian or Alaska Native, and 1% Arab, Middle Eastern, or North African). Four culturally relevant school support profiles were identified: (1) *low general, devoid cultural & critical support;* (2) *moderate general, moderate cultural, & devoid critical support;* (3) *high general, moderate cultural & critical support;* and (4) *high general, cultural, & critical support*. Youth in the *high general, cultural, & critical support* profile had higher concurrent emotional and behavioral engagement. No significant differences were found between early (6^th^ grade) and middle adolescent (9^th^ grade) youth, pointing to the relevance and associations of the identified profiles across development. One significant difference emerged when comparing White and ethnoracially minoritized youth; among White youth, those in the *high general, cultural, & critical support* and *high general, moderate cultural & critical support* profiles had higher academic expectations than those in the *low general, devoid cultural & critical support* profile. The discussion focuses on characterizing heterogeneous and culturally relevant school support profiles, the associations between these profiles and indicators of academic functioning for ethnoracially diverse youth to advance developmental theory and the importance of promoting culturally relevant school support practices to foster developmental competencies among youth.

## Introduction

Youth are developing in the ecological context of a rapidly diversifying world. As U.S. and global societies experience increasing ethnic-racial diversity (Pew Research Center, 2019, April 22; U.S. Census Bureau, 2021, August 12), schools have an opportunity to adapt to these changes by supporting youth development to meet their needs of engaging with and shaping a multicultural society. Although researchers have documented the positive implications of various dimensions of teacher support and school practices that affirm the child and their home communities for academic functioning (Dee & Penner, 2017; Tao et al., 2022), recent theoretical advancements support the importance of considering multiple culturally relevant dimensions for all youth (Wantchekon & Umaña-Taylor, 2024). However, research to date has not examined how youth’s heterogeneous experiences of culturally relevant support dimensions in school, such as *critical consciousness socialization*, *cultural competence development*, and *teacher support*, jointly relate to youth academic functioning (i.e., academic aspirations, expectations, and engagement). Thus, the present study focuses on the following aspects of school support: (1) *teacher support* (herein also termed general support) that involves helpfulness and availability of support from teachers as perceived by students (Torsheim et al., 2000), (2) *promotion of cultural competence* that includes learning about ethnoracial group histories and traditions other than one’s own (Hernández et al., 2023), and (3) *critical consciousness socialization* that includes learning about power, privilege, and systems of oppression (Diemer et al., 2016). These culturally relevant school support dimensions have been examined in isolation from one another, even though these forms of support are theorized to *jointly* shape adolescent academic outcomes (Ladson-Billings, 1995). Addressing this omission requires adopting a holistic and comprehensive view of adolescent experiences as a learner and a cultural being (García Coll et al., 1996) to characterize various constellations of culturally relevant school support using a person-centered approach (Suzuki et al., 2021). Thus, the purpose of this study was to examine profiles based on adolescent experiences of general, cultural competency, and critical reflection support in school to help us understand variability in students’ encounters with culturally relevant school practices that might differentially promote their academic development.

### A Person-Centered Approach and Theory on Culturally Relevant School Support

According to theory on culturally relevant pedagogy (Ladson-Billings, 1995; 2004), teachers who engage in culturally relevant practices foster a sense of connectedness via *teacher support*, hold their students to high academic and behavioral expectations, center youth’s cultural lives to make learning effective, and promote youth understanding of ethnic-racial identities, inequalities, and social justice issues through the *promotion of cultural competence* and *critical consciousness socialization* (Byrd, 2016). These tenets of culturally relevant pedagogy and its implementation across school subjects align with the emphasis on promoting school environments, including relationships with teachers (García Coll & Szalacha, 2004), and youth cultural strengths and competencies advanced by foundational models of minority youth development, including the Integrative Model for the Study of Developmental Competencies (García Coll et al., 1996). Accordingly, culturally relevant practices are theorized to be proximal mechanisms through which schools become supportive contexts that foster youth’s psychological and academic functioning (García Coll et al., 1996). Relatedly, recent research on school ethnic-racial socialization, which includes cultural socialization (Huguley et al., 2019), also proposes that schools are sites where youth learn about their own and others’ ethnic-racial identities (Saleem & Byrd, 2021), which form a basis for promoting the holistic development and academic achievement of youth from all ethnic-racial backgrounds. Thus, culturally relevant school support requires culturally engaged (e.g., promoting multicultural skills), critically reflective (e.g., critical consciousness socialization), and general support (e.g., teacher support) that validates students’ experiences.

Building on this work, culturally relevant school support is conceptualized to be multi-dimensional and involve teacher support, critical consciousness socialization, and the promotion of cultural competence in school. The promotion of cultural competence fosters multi-cultural competencies, critical consciousness socialization provides youth with opportunities for reflection and meaning-making of their cultural and racialized experiences (Diemer et al., 2016; Mathews et al., 2020), and general teacher support contributes to a supportive school environment (Torsheim et al., 2000). General teacher support, although not a culturally engaged or critically specific support source, is, nonetheless, a key culturally relevant dimension of student support (Ladson-Billings, 1995). In the absence of critical or cultural support, general teacher support does not respond to the needs of youth to make sense of an increasingly diversifying and socially stratified society. In the absence of general teacher support, critical or cultural support might not be perceived by youth as authentic or caring. In the presence of cultural support but not critical support, youth might not be as equipped to address the social inequities they confront. Thus, experiencing highly culturally relevant support requires high levels of critical consciousness, cultural (e.g., promoting multicultural skills), and general teacher support. Currently, there is limited understanding of how youth experience various constellations of general, critical, and cultural support from their teachers and the distinct associations between the patterning of such support and academic functioning.

Addressing this gap and identifying how different profiles of support in school are associated with informing adolescent academic functioning necessitates adopting a person-centered – rather than variable-centered -- approach due to its conceptual and analytical advantages (Suzuki et al., 2021). At a conceptual level, a person-centered approach facilitates the identification of the above noted distinct constellations of support that youth receive in their schools. Next, it is consistent with antiracist developmental science (Suzuki et al., 2021) and the integrative model of minoritized youth development (García Coll et al., 1996) because the present study’s person-centered approach seeks to describe heterogeneity by identifying subgroups as a function of multiple types of school support youth receive rather than ethnoracial group membership, which allows for a more holistic view on the complexities of adolescent lived experiences (e.g., Byrd & Ahn, 2020). From an analytical standpoint, the person-centered approach implemented as a latent profile analysis is advantageous over variable-centered approaches because of higher statistical power to detect effects, ability to detect nonlinear effects, and moving away from testing three-way (or higher) interaction terms (Meyer & Morin, 2016). Thus, the present study uses a person-centered approach to identify profiles of student perceived general, cultural competence, and critical school support and to examine how belonging to such profiles is associated with academic outcomes.

### Associations between Culturally Relevant Support Profile Dimensions and Academic Functioning

Encountering and engaging with culturally relevant and multicultural practices in school is theorized to support youth psychological adjustment (Barrett, 2018) and academic functioning (Graham, 2018). However, the research literature has rarely focused on capturing multiple culturally relevant school practices and their link with youth academic functioning (see Byrd & Ahn, 2020, for an exception). Thus, the present study draws on evidence on individual culturally relevant support dimensions (i.e., teacher support, promotion of cultural competence, critical consciousness socialization) and their links with academic functioning to establish the empirical foundation and subsequently move towards a multi-dimensional conceptualization of school support profiles as outlined by culturally responsive theories (Ladson-Billings, 1995).

Considering general teacher support, research to date has documented positive associations, albeit of small to moderate magnitude, between general support and measures of adolescent academic functioning (Gale, 2020; Givens Rolland, 2012; Tao et al., 2022). Self-determination theory posits that overall teacher support fulfills students’ need for relatedness and promotes a learning environment where students are engaged and motivated to do well in school (Deci & Ryan, 2000). Evidence from meta-analyses indicates that teacher support is a consistent predictor of school belonging (Allen et al., 2016), academic engagement (Roorda et al., 2019), and achievement (Tao et al., 2022). This body of evidence suggests that teacher support predicts positive academic functioning, whereas feeling disconnected and unfairly treated by teachers increases the risk of poor academic functioning. Perceptions of general teacher support typically decline over adolescence (Castro-Schilo et al., 2016), which presents a risk for academic functioning declines in middle and high school.

The promotion of cultural competence, a theorized cultural support mechanism of school ethnic-racial socialization, fosters improved inter-group attitudes and interactions (Verkuyten & Thijs, 2013) and enhances academic outcomes for both minoritized (Saleem & Byrd, 2021) and White youth (Satterthwaite-Freiman & Umaña-Taylor, 2024). Schools can promote multicultural competence – the ability to interact and create connections with people from different cultural backgrounds – by providing time and space, physical and educational, to teach about ethnic-racial groups and their histories. In teaching about diverse cultures, races, and traditions, schools are signaling to students that the histories and traditions of various communities are worth learning about.

Evidence has linked teachers’ promotion of cultural competence with various positive academic outcomes, including school belonging, academic motivation, retention rates, and grades (Byrd, 2015; Byrd, 2016; Byrd & Chavous, 2011; Del Toro & Wang, 2021; Tan, 2001). For instance, cultural competence was positively associated with school belonging and academic self-concept, motivation, and achievement among immigrant and non-immigrant German students (Schachner et al., 2019). Similar findings have been documented among those from minoritized backgrounds, particularly among Black and Latinx youth (Byrd & Hope, 2020). For example, Latinx students who received instruction that promoted cultural competencies reported easier learning and better grades and believed they would graduate from high school (Tan, 2001). School cultural competence is expected to positively impact Latinx academic achievement by promoting ethnocultural empathy (Chang & Le, 2010). Although this body of evidence underscores the presence and promotive nature of teacher’s engagement in critically conscious and culturally specific support for adolescent academic functioning, recent focus groups with African American and Latinx youth reveal high frequency but somewhat limited content of critically promotive messages from their educators (e.g., specific historical events and figures; Byrd & Hope, 2020; Sladek et al., 2022). Thus, there appears to be heterogeneity in exposure and messages that youth receive from teachers aimed at promoting cultural competence that might not be accompanied by critical consciousness support, validating the premise and need for the present study’s more detailed, person-centered look at these processes.

Critical consciousness socialization is among the key mechanisms of ethnic-racial socialization through which schools and educators contribute to adolescent development (Saleem & Byrd, 2021). Critical consciousness has three components: *critical reflection* involves becoming aware of social inequities (e.g., racism, classism, sexism); *sociopolitical efficacy* reflects one’s motivation and self-efficacy to enact sociopolitical change; and *critical action* involves engaging in acts to disrupt social inequity and promote social justice (Heberle et al., 2020). The present study focuses on teachers’ and schools’ socialization of critical reflection. When educators engage in socialization practices to foster critical consciousness, they promote awareness of race, racism, and civic development (Saleem & Byrd, 2021). In general, critical consciousness socialization is theorized to promote racially marginalized youth and White youth (Heberle et al., 2020) development by encouraging reflection and making meaning of youth’s racialized experiences (Diemer et al., 2016; Mathews et al., 2020).

Critical consciousness development has been linked to positive academic outcomes. Pedagogy that leads to increased critical consciousness has been associated with higher student engagement and improved standardized test scores among students from a low-performance middle school (Luter et al., 2017). In a similar vein, in a five-year longitudinal quasi-experimental study in which students below a grade point average (GPA) threshold were assigned to an Ethnic Studies course focused on the teaching of historic social and political struggles of multiple minoritized groups (e.g., the genocide of Native Americans in California), students who initially struggled academically and participated in the Ethnic Studies course showed a substantial increase of 16–19% in graduation rates (Bonilla et al., 2021). Also, adolescents’ critical reflection and critical action predicted higher standardized test scores, whereas critical action predicted higher GPAs (Seider et al., 2020). The positive association between critical consciousness development and academic achievement may be stronger for students of color than their White counterparts (Seider et al., 2023). This accumulating evidence underscores the need for continued examination of the impact of school critical consciousness socialization on adolescent academic outcomes and the need to consider possible ethnic-racial differences.

To date, only limited research has characterized the multiple dimensions of culturally relevant support in school and their links with academic functioning. In a person-centered study of school ethnic-racial discrimination (which would represent low teacher support) and ethnic-racial socialization, one study found three profiles (average, high discrimination, and positive school; Byrd & Ahn, 2020). Unfair treatment in school based on reports of discrimination distinguished a “high discrimination” from a “positive school” profile; youth in a high discrimination profile experienced the highest ethnic-racial socialization messages and racial discrimination in school, whereas those in a positive school profile experienced high ethnic-racial socialization messages and low racial discrimination. Those in the positive school profile had better academic functioning than the remaining two profiles. These findings did not differ across grades or ethnic-racial backgrounds. When teachers delivered an ethnic-racial identity development school intervention, as an example of culturally and critically informed support, and provided emotional support to their students (akin to general teacher support), youth reported higher levels of academic engagement (Wantchekon & Umaña-Taylor, 2024). This finding emphasizes the need to consider and examine how multiple indicators of school culturally relevant support practices collectively relate to academic functioning. Given that social positionality (García Coll et al., 1996), family support (Wang & Eccles, 2012), and academic experiences (Hernández et al., 2016) could inform culturally relevant school support patterns or academic functioning, the present study also examined how these key covariates (i.e., ethnic-racial background, gender, family socioeconomic status, family social support, and prior GPA) predicted the profiles.

### Developmental Period and Ethnoracial Moderators

#### Developmental period

Evidence suggests that ethnic-racial identity exploration and resolution increase from early to late adolescence due to enhanced cognitive skills (Umaña-Taylor, 2018). However, very few studies have examined whether patterns and determinants of culturally relevant experiences in school vary by age. Increased salience of ethnic-racial identity from early to middle adolescence suggests that compared to younger youth, older youth might be more interested in and attuned to culturally relevant practices, including interpreting concepts related to race (Saleem & Byrd, 2021). However, one study found that adolescents in a positive school group (comprised of youth with high ethnic-racial socialization messages and low racial discrimination and critical consciousness) were significantly younger than those in average (youth with low to moderate levels of ethnic-racial socialization messages) or high discrimination (youth with the highest ethnic-racial socialization messages, critical consciousness, and racial discrimination in school) profiles (Byrd & Ahn, 2020). Thus, adolescents might be more likely to experience relatively more positive ethnic-racial socialization in school at younger than later ages, but also be more attuned to unfair treatment as they accumulate experiences and develop a more critical awareness of potential differential treatment based on ethnic-racial backgrounds.

In terms of developmental differences in the associations of culturally relevant school support on academic functioning, very few empirical studies have examined this possibility. A recent meta-analysis tested and found that school multicultural climate (comprised of measures related to learning about different cultures as well as one’s own) was significantly associated with multiple outcomes (among them academic measures) for secondary but not primary school students (Bardach et al., under review). Thus, because older youth might be more attuned to culturally relevant support (Saleem & Byrd, 2021), older youth might be more ready to benefit from learning about and engaging in culturally relevant practices, suggesting that the associations between culturally relevant school support profiles and academic functioning might be stronger among middle adolescents (in 9^th^ grade) than early adolescents (in 6^th^ grade).

#### Ethnoracial status

Consistent with the notions introduced above, the associations between culturally relevant school support profiles and academic functioning might be stronger among ethnoracial minoritized youth than White youth, given the increased salience of ethnic-racial identity for ethnoracial minoritized youth (Umaña-Taylor, 2018). In addition, some research suggests that the teacher-student relationship quality is more closely intertwined with cultural and critical support indicators of culturally relevant support among ethnoracial minoritized youth compared to majority youth (Paizan et al., 2024). Studies testing whether culturally relevant school support experiences and their associations vary across youth from different ethnic-racial backgrounds are scarce. One person-centered study found that White students were overrepresented and Black students were underrepresented in a positive school profile with high ethnic-racial socialization messages and low racial discrimination and critical consciousness (Byrd & Ahn, 2020), suggesting that White youth might experience relatively positive ethnic-racial socialization messages but lower critical consciousness socialization than ethnoracially minoritized youth. In another study of fifth grade students, school cultural socialization was significantly and positively associated with behavioral and affiliative academic engagement for African American youth but not for White youth (Del Toro & Wang, 2021). Although few studies have tested this supposition, it is plausible that the association between culturally relevant school support and academic functioning might be stronger among ethnoracially minoritized youth compared to White youth.

## Current Study

Research examining general teacher support has rarely also considered student perspectives on cultural socialization and critical consciousness support in school, warranting a need for collectively examining these key support dimensions among youth. The current study identified adolescent culturally relevant school support profiles based on a person-centered analysis of student-reported levels of teacher support, promotion of cultural competence, and critical consciousness socialization among ethnoracially minoritized and White youth (Aim 1). The presence of four culturally relevant school support profiles with varied levels across three culturally relevant support dimensions was expected: high general, cultural, and critical support (e.g., high teacher support, promotion of cultural competence, critical consciousness socialization), low general, cultural, and critical support (e.g., low teacher support, promotion of cultural competence, critical consciousness socialization), devoid cultural and critical support (e.g., high to moderate teacher support but relatively lower promotion of cultural competence and critical consciousness), and devoid critical support (e.g., moderate teacher support, relatively moderate to lower promotion of cultural competence, and low critical consciousness socialization). This study also examined how key indicators of social position (i.e., ethnic-racial background, gender, family socioeconomic status, family social support, and prior GPA) predicted the profiles and how these profiles inform youth academic functioning based on academic behavioral and emotional engagement, and academic aspirations and expectations accounting for covariates (Aim 2). It was hypothesized that experiencing high general, cultural, & critical support (i.e., high teacher support, critical consciousness socialization, promotion of cultural competence) would be associated with higher academic functioning among youth, whereas experiencing low support across culturally relevant support dimensions will be associated with low academic functioning. It was also hypothesized that youth who experience devoid cultural and critical support (e.g., high to moderate teacher support but relatively lower promotion of cultural competence and critical consciousness) would experience higher academic functioning than those with devoid critical support (moderate teacher support, relatively moderate to lower promotion of cultural competence, and low critical consciousness socialization), but lower academic functioning than those with high general, cultural, and critical support. For instance, a person experiencing high levels of teacher support might confer academic benefits from receiving generalized support from teachers but might not reach their full academic potential if they experience low levels of critical consciousness socialization and promotion of cultural competence in school, given that adolescence is a salient period for ethnic-racial identity development, particularly in multicultural environments. As an exploratory Aim 3, multiple group analyses were conducted to test for possible developmental and ethnic-racial differences in the pattern of results. It was predicted that the hypothesized profiles would be most strongly associated with academic functioning for ethnoracially minoritized youth (compared to White youth) and youth in 9^th^ grade (compared to 6^th^ grade).

## Methods

### Participants

The sample included 717 students (49.9% girls; *M*_age-years_ = 13.73, *SD* = 1.54, range: 10–18 years) in 6^th^ (*n* = 280) and 9^th^ (*n* = 437) grades from two middle and two high schools from a public school district in U.S. Southwest. Participant’s self-reported race/ethnicity was 31.8% Hispanic/Latinx, 31.5% Multiethnic, 25.7% White, 7.3% Black or African American, 1.4% Asian American or Pacific Islander (AAPI), 1.4% American Indian or Alaska Native (AI/AN), and 1% Arab, Middle Eastern, or North African (AMENA). Participants of Hispanic/Latinx heritage specified being of Mexican origin (89.5%), Puerto Rican (2.5%), Salvadoran (1%), and another origin (6.8%). Moreover, 66% of participants were 3^rd^ generation (both youth and parents born in the U.S.), 17.7% were 2.5 generation (one parent born abroad and youth and one parent born in the U.S.), 11.8% were 2^nd^ generation (youth born in the U.S. and both parents born abroad), and 4% were 1^st^ generation (youth and parents born abroad).

For parents’ educational levels, participants reported that 10.4% of mothers had less than a high school diploma, 21.3% finished high school or GED, 8.4% went to community college or trade school, 21.8% had completed some college, 17.1% had a college degree, and 20.9% had a professional degree (MA, PhD, JD, MD). For paternal education, 15% had less than high school, 31.4% had a high school diploma or GED, 7.5% had an associate degree, 18.5% had completed some college, 12% had a college degree, and 15.7% had a professional degree (MA, PhD, JD, MD). In terms of socioeconomic status, 24.3% of participants reported that they never had to worry about money, 38.2% stated that their family only had to worry about money for fun and extras, 35.1% reported they had just enough to get by, and 2.3% indicated that they did not have enough to get by. Thirty-nine percent of participants reported receiving free or reduced-price lunch at school.

### Procedure

Students from all four schools received parental consent letters in English and Spanish and received $10 for returning their signed consent form regardless of parental decision on their participation in the study. Teachers also received an incentive of $50 and two movie tickets to remind students to return signed forms to school. Participants provided their assent prior to completing their surveys. These procedures were approved by the Arizona State University and school district institutional review boards. Participants completed self-reported questionnaires in English during their regular school hours over two class periods (approximately 90 min total) between December 2019 and early January 2020. School staff and research assistants were available to answer questions as participants completed the survey. School GPA records were collected for the fall semester of 2019.

### Measures

#### Culturally relevant school support: teacher support

Youth reported on teacher support (4 items, α = .86, e.g., “My teachers are interested in me as a person,” see Appendix A) using the Teacher and Classmate Support Scale (Torsheim et al., 2000). Prior work has supported the validity and reliability of these subscales among ethnoracially diverse adolescent samples (Fernández-Lasarte et al., 2019). Validation findings confirm the construct validity of the subscale (Torsheim et al., 2000). Youth were asked to reflect on the past 6 months and respond to how strongly they agreed with each statement on a scale from 1 (*strongly disagree*) to 5 (*strongly agree*). The original scale directions had 12 months as a time reference; this study used 6 months as a reference, corresponding to the data collection being approximately the midpoint of the school year. Perceived teacher support was calculated from the average of all items.

#### Culturally relevant school support: critical consciousness socialization and promotion of cultural competence

Youth reported on items from the critical consciousness socialization (4 items, α = .87, e.g., “Your teachers encourage awareness of social issues affecting your culture”) and promotion of cultural competence subscales (5 items, α = .92, e.g., “At your school, they encourage you to learn about different cultures”), both from the School Climate for Diversity - Secondary scale (SCD-S; Byrd, 2017) used in prior research. The responses used a 5-point Likert-type scale ranging from 1 (*not true at all*) to 5 (*completely true*). Prior work has supported the validity and reliability of these subscales among ethnoracially minoritized adolescent samples (Byrd, 2017). Mean scores were calculated, with higher scores indicating greater critical consciousness socialization and promotion of cultural competence.

#### Academic functioning: academic aspirations and expectations

Youth reported their academic aspirations (“How far would you like to go in school?”) and expectations (“How far do you really think you will go in school?). Responses ranged: 1 = *some high school*, 2 = *high school graduate or GED*, 3 = *some college but no degree*, 4 = *graduate from a 2-year college, vocational, or technical school, or join the military*, 5 = *graduate from a four-year college*, 6 = *get an MS/MA*, and 7 = *get a professional degree*). Students aspired to attain between a 4-year and a Master’s degree (*M* = 5.24, *SD* = 1.55) and expected to attain between a 2- and 4-year degree (*M* = 4.47, *SD* = 1.66).

#### Academic functioning: behavioral and emotional academic engagement

Youth behavioral and emotional academic engagement was measured using subscales from the Engagement versus Disaffection with Learning Scale (Skinner et al., 2009). Participants reported on behavioral (6 items; α = .91; e.g., “When I am in class, I participate in class discussions”) and emotional academic engagement (4 items; α = .90; e.g., “I enjoy learning new things in class”). Prior work has supported the validity and reliability of these subscales among ethnoracially diverse adolescent samples (e.g., Martinez-Fuentes et al., 2021), including strong correlations between student and teacher reports and observations of academic engagement (Skinner et al., 2009). Responses ranged from 0 (*never*) to 4 (*all the time*). Higher mean scores indicated higher behavioral and emotional academic engagement.

#### Covariates

Covariates included youth reports of their subjective appraisal of family socioeconomic status (i.e., youth response to “How much money does your family have?” [1 = *Not enough to get by*, 2 = *Just enough to get by*, 3 = *We only have to worry about money for fun and extras*, 4 = *We never have to worry about money*]), perceived family social support (4 items, α = .90, Multidimensional Scale of Perceived Social Support; Zimet et al., 1988), gender (1 = *girl*; 0 = *boy*; other cases were coded as missing), and ethnicity-race (1 = *ethnoracially minoritized*; 0 = *non-Hispanic White*). Students who chose to identify with multiple ethnic-racial categories were coded as Multiethnic; youth from Hispanic/Latinx, Multiethnic, Black/African American, AAPI, AI/AN, and AMENA backgrounds were coded as ethnoracially minoritized. School records of grade (1 = *ninth*; 0 = *sixth*) and GPA for the fall semester of 2019 were used as covariates.

### Data Analysis Plan

Descriptive statistics and bivariate correlations among study variables were examined prior to conducting multivariate analyses. The study’s aims were estimated with latent profile analyses (LPA) in *M*plus 8.1 (Muthén, 2004). Across study variables, there were 0% to 14% missing values. Because imputation is not appropriate for analytical approaches such as LPA, which assumes multiple underlying populations, full-information maximum likelihood (FIML) was used to handle missing data. Possible changes in profile composition with the use of FIML were monitored when covariate and distal outcome variables were estimated in the models.

A person-centered approach (Suzuki et al., 2021) was used to estimate the latent profiles of adolescents’ experiences of school support using average scores of their reports of teacher support, school promotion of cultural competence, and critical consciousness socialization as indicators. Diagonal class-varying models were estimated, which allow for variances to be free to vary across classes, and covariances between indicators are fixed at zero within classes. Solutions with up to five profiles were examined, and the best-fitting model was selected based on the following criteria: smaller Akaike Information Criterion (AIC; Bozdogan, 1987), Bayesian Information Criterion (BIC; Schwarz, 1978), sample-size adjusted BIC (aBIC; Sclove, 1987), and approximate weight of evidence (AWE; Banfield & Raftery, 1993); a significant bootstrapped likelihood ratio test (BLRT; Masyn, 2013) and Vuong-Lo-Mendell-Rubin likelihood ratio test (VLMR; Lo et al., 2001; Vuong, 1989) for model K and non-significant BLRT and VLMR for model K + 1; a relatively large approximate correct model probability (c*m*P; Masyn, 2013) indicating the probability of a given model being correct out of all fitted models; appraisal of the smallest profile size (Ferguson et al., 2020); and conceptual interpretability of the profiles (Tofighi & Enders, 2008). Entropy was evaluated as a measure of class categorization (Nylund-Gibson & Choi, 2018).

After estimating the profiles, tests of measurement equivalence in the profiles based on developmental (between grades) and ethnic-racial backgrounds were tested (Morin et al., 2015; Olivera-Aguilar & Rikoon, 2017). Evidence for at least structural equivalence (i.e., equal profile means between groups) was necessary to test potential predictive and explanatory differences with multiple group analyses (exploratory Aim 3).

Then, associations between background characteristics (i.e., family socioeconomic status, gender, family social support, prior GPA) and the latent profile solution were examined using the manual three-step approach (Asparouhov & Muthén, 2014; Vermunt, 2017). This approach estimates multinomial logistic regressions assessing the probability of being in one profile over another. The profile solution was regressed on background characteristic variables while accounting for profile classification error. Odds ratio estimates indicate how each background characteristic relates comparatively to the estimated profiles. Predictive differences in the associations between background characteristics and the latent profile solution were tested to determine if there was evidence for measurement equivalence in the profiles based on grades and ethnic-racial backgrounds.

The associations between the identified latent profile solution and youth academic functioning (i.e., academic aspirations and expectations, emotional and behavioral academic engagement) were examined using the manual three-step approach, in which both background characteristic predictors and distal outcomes of the latent profiles were estimated (Asparouhov & Muthén, 2014; Bakk & Vermunt, 2015; Nylund-Gibson et al., 2019). Cases in which adolescents identified as a gender other than boy or girl (1.1%) were omitted from these analyses due to the small sample size. Pairwise z-tests were used to test significant mean differences in the academic functioning outcomes across the profiles for each set of profile pairs (e.g., Profiles 4 versus 1) while accounting for classification error. If there was evidence for measurement equivalence in the profiles based on grades and ethnic-racial backgrounds, explanatory differences in the associations between the latent profile solution and youth academic functioning were tested.

## Results

Table 1 includes correlations and descriptive statistics for the study variables. Academic aspirations, academic expectations, and behavioral engagement scores were positively correlated with one another. Teacher support, school promotion of cultural competence, and critical consciousness socialization in school were also correlated with one another. Teacher support, school promotion of cultural competence, and critical consciousness socialization were positively correlated with behavioral and emotional engagement, whereas teacher support was positively correlated with academic aspirations and expectations.

**Table 1:** Correlations and descriptive statistics

		1	2	3	4	5	6	7	8	9	10	11	12	13
Academic functioning														
1.	Academic aspirations	--												
2.	Academic expectations	0.71***	--											
3.	Behavioral engagement	0.18***	0.32***	--										
4.	Emotional engagement	0.08*	0.18***	0.67***	--									
School support practices														
5.	Teacher support	0.07*	0.11**	0.31***	0.44***	--								
6.	Promotion of cultural competence	0.02	0.04	0.33***	0.41***	0.33***	--							
7.	Critical consciousness socialization	0.00	0.04	0.28***	0.39***	0.33**	0.71***	--						
Covariates														
8.	Family SES^a^	0.12**	0.16***	0.14***	0.09*	0.05	0.09*	0.11**	--					
9.	Family social support	0.09*	0.18***	0.25***	0.27***	0.25***	0.18***	0.20***	0.17***	--				
10.	GPA (Fall 2019)^b^	0.37***	0.43***	0.39***	0.23***	0.19***	0.08*	0.06	0.18***	0.22***	--			
11.	Ninth grade^c^	−0.01	0.06	−0.16***	−0.18***	−0.14***	−0.20***	−0.17***	0.01	−0.07	−0.10**	--		
12.	Girl^d^	0.20***	0.11**	−0.02	−0.13***	−0.09*	−0.09*	−0.09*	−0.04	−0.08*	0.11**	0.04	--	
13.	Ethnoracial minority^e^	−0.03	−0.10*	−0.06	−0.02	0.01	0.01	0.03	−0.15***	−0.08*	−0.15***	−0.23***	0.03	--
M/Frequency		5.24	4.47	2.87	2.31	3.68	3.13	2.91	2.85	5.27	2.74	0.61	0.51	0.74
SD		1.55	1.66	0.70	0.87	0.83	1.02	1.09	0.82	1.61	0.97	0.49	0.50	0.44
*N*		690	687	710	709	710	694	689	646	615	704	717	699	717
% of cases		96%	96%	99%	99%	99%	97%	96%	90%	86%	98%	100%	97%	100%

^a^Youth-perceived family socioeconomic status

^b^Grade point average assessed in the fall semester of 2019

^c^Ninth grade (1 = ninth grade; 0 = sixth grade)

^d^Girl (1 = girl; 0 = boy)

^e^Ethnoracial minority (1 = ethnoracial minoritized; 0 = non-Hispanic White)

*p < .05, **p < .01, ***p < .001

### Identifying and Predicting School Support Profiles Based on Culturally Relevant Support Dimensions

In testing Aim 1, a four-profile LPA solution was selected because it was the best-fitting model with good interpretability based on theory (see Table 2; Byrd, 2016; Ladson-Billings, 1995, 2004). Although the five-profile solution had lower AIC, BIC, adjusted BIC values, and a higher c*m*P value than all the other solutions, one of the identified profiles was considerably below the recommended minimum proportion at 2% (Ferguson et al., 2020). Compared to the solutions with one, two, and three profiles, the four-profile solution had lower AIC, BIC, adjusted BIC values, and a higher c*m*P value. In addition, the AWE criterion was highest for the four-profile solution compared to all the other solutions, and the VLMR was significant for a four-profile solution and non-significant for a five-profile solution. Of note, all the estimated profile solutions had statistically significant BLRT values, which are sensitive to the addition of parameters, and therefore, a change from significant to non-significant value was not used as a criterion to determine the best-fitting profile solution when comparing two models; rather, relative model improvement based on additional parameters was evaluated (Ferguson et al., 2020).

**Table 2:** Model fit criteria for latent profile analysis of culturally relevant support practices in school (*n* = 712)

Index	1 profile	2 profiles	3 profiles	4 profiles	5 profiles
Loglikelihood	−2908.43	−2704.90	−2574.98	−**2505.47**	−2461.14
Parameters	6	13	20	**27**	34
AIC	5828.86	5435.79	5189.96	**5064.95**	4990.29
BIC	5856.27	5495.18	5281.32	**5188.29**	5145.60
aBIC	5837.22	5453.90	5217.81	**5102.56**	5037.64
AWE	5913.68	5619.56	5472.68	**5446.62**	5470.92
Entropy	1.00	.79	.85	**.81**	.88
Δ AIC	--	−393.07	−245.84	−**125.01**	−74.66
Δ BIC	--	−361.09	−213.86	−**93.03**	−42.69
Δ aBIC	--	−383.32	−236.09	−**115.26**	−64.91
Δ AWE	--	−294.12	−146.88	−**26.05**	24.29
BLRT	--	<.001	<.001	**<.001**	<.001
VLMR		<.001	<.001	**.02**	.73
c*m*P	4.79E-155	1.23E-76	3.38E-30	**5.38E-10**	1.00
Smallest profile (% of sample)^a^		185 (26%)	66 (9%)	**52 (7%)**	12 (2%)

Lower AIC, BIC, aBIC, and AWE values represent better fit. A larger c*m*P value represents a larger probability of a given model is correct out of all fitted models. A significant BLRT for model K and non-significant BLRT for model K + 1 indicates that the model with K profiles is a better fitting model. Boldface indicates the solution that was selected as the best fitting model

*AIC* Akaike Information Criterion, *BIC* Bayesian Information Criterion, *aBIC* adjusted BIC, *AWE* approximate weight of evidence, *BLRT* bootstrapped likelihood ratio test, *VLMR* Vuong-Lo-Mendell-Rubin likelihood ratio test, *cmP* approximate correct model probability

^a^Counts and proportions for the smallest latent profile are based on the estimated model’s probabilistic likelihood of profile membership

**Fig. 1:**
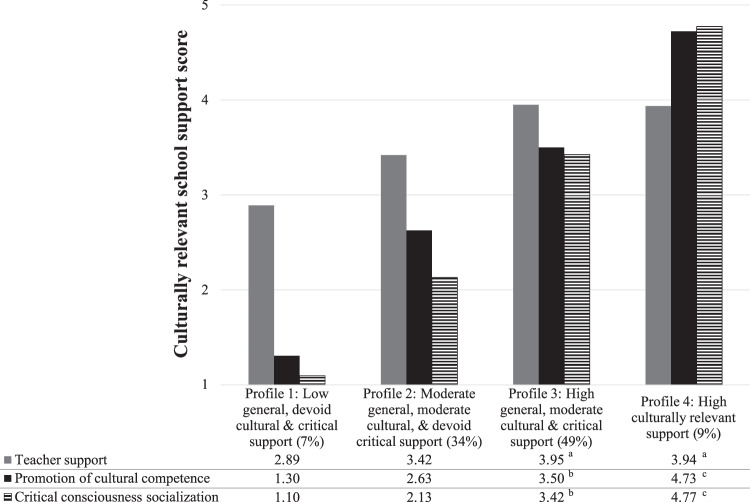
Aim 1: Means and Proportions of Culturally Relevant School Support Latent Profiles. Note. Average culturally relevant school support scores are depicted under each profile label. Profile indicator scores were tested for equivalence across and within profiles. Scores with the same superscript letter (**a**, **b**, **c**) represent equivalent scores based on Wald-tests of equivalence and all other scores were significantly different from each other (*p* < .05). Latent profile proportions are based on the classified profile membership final count

Aligned with the study’s hypotheses, the four identified profiles were characterized by different relative levels of culturally relevant school support (Fig. 1). In this model, a *low general, devoid cultural & critical support* (#1) profile (*n* = 52; 7%) had distinctly low promotion of cultural competence and critical consciousness socialization scores (almost two *SD*s below the sample means) and low teacher support (about one *SD* below the sample mean). The *moderate general, moderate cultural, & devoid critical support* (#2) profile (*n* = 245; 34%) had slightly moderate teacher support (about one-third *SD* below the sample mean), moderate promotion of cultural competence (0.5 *SD* below the sample mean), and low cultural consciousness socialization scores (0.7 *SD* below the sample mean). The first two profiles had similar differences across culturally relevant school support dimensions, such that teacher support was significantly higher than both critical and culturally specific support measures, and promotion of cultural competence was significantly higher than critical consciousness socialization. The remaining two profiles had high teacher support and varying levels of culturally specific support across profiles (although similar scores within profiles). For instance, the *high general, moderate cultural & critical support* (#3) profile (*n* = 351; 49%) had high teacher support (about one-third *SD* above the sample mean) and moderate cultural and critically specific support (from 0.4 to 0.5 *SD*s above the sample mean). The *high general, cultural, & critical support* (#4) profile (*n* = 64; 9%) had high teacher support (about one-third *SD* above the sample mean) and the highest cultural and critically specific support (about 1.6 to 1.7 *SD*s above the sample mean). The majority of adolescents (83%) were in the *moderate general, moderate cultural, & devoid critical* (#2) and *high general, moderate cultural & critical* (#3) support profiles, whereas fewer were in the *low general, devoid cultural & critical* (#1) and *high general, cultural, & critical* (#4) support profiles, suggesting that few adolescents in the study’s sample experienced very low or high levels of support in school.

To test for predictive and explanatory differences across grades and ethnicity-race (exploratory Aim 3), measurement equivalence was first tested (Table 3). There was evidence for distributional similarity across ethnicity-race (i.e., the profile means, variances, and distributional sample sizes were equal between ethnoracially minoritized and White youth). Based on evidence of dispersion similarity between grades (i.e., the profile means and variances were equal between sixth and ninth graders), the *low general, devoid cultural & critical* (#1) and *moderate general, moderate cultural, & devoid critical* (#2) support profiles had proportionally more 9^th^ graders than 6^th^ graders. The *high general, moderate cultural & critical* (#3) and *high general, cultural, & critical* (#4) support profiles had proportionally more 6^th^ graders than 9^th^ graders.

**Table 3:** Model fit for latent profile multiple group comparisons across grades and ethnoracial background

Model	BIC	aBIC	Δ BIC	Δ aBIC	LL	#fp	MLR cf	cd	Δ LL	Δ df	*p*
**Comparisons between 6**^**th**^ **and 9**^**th**^ **grade youth**											
Measurement equivalence											
Configural (unconstrained)	6288.21	6113.57			−2963.48	55	1.21				
Structural (equal means)	6209.86	6073.33	−78.34	−40.24	−2963.72	43	1.25	1.10	0.43	12	1.00
Dispersion (equal means and variances)	6135.93	6037.50	−73.93	−35.83	−2966.16	31	1.21	1.34	3.64	12	.99
Distributional (equal means, variances, probabilities)	6148.32	6059.42	12.39	21.92	−2982.21	28	1.24	0.96	33.46	3	< .001
Predictive similarity											
Freely estimated	11715.86	11569.80			−5707.29	46	1.05				
Equally estimated	11633.65	11535.22	−82.20	−34.58	−5715.31	31	1.04	1.07	14.94	15	.46
Explanatory similarity: Emotional engagement											
Freely estimated	13335.15	13185.91			−6513.66	47	1.05				
Equally estimated	13313.92	13177.39	−21.22	−8.52	−6516.14	43	1.06	1.02	4.89	4	.30
Explanatory similarity: Behavioral engagement											
Freely estimated	13083.93	12934.70			−6388.05	47	1.11				
Equally estimated	13063.65	12927.12	−20.28	−7.58	−6391.01	43	1.07	1.51	3.92	4	.42
Explanatory similarity: Academic aspirations											
Freely estimated	14228.51	14079.28			−6960.34	47	1.03				
Equally estimated	14204.35	14067.82	−24.16	−11.46	−6961.36	43	1.00	1.26	1.62	4	.81
Explanatory similarity: Academic expectations											
Freely estimated	14307.46	14158.22			−6999.81	47	0.98				
Equally estimated	14290.91	14154.37	−16.55	−3.85	−7004.64	43	0.96	1.10	8.76	4	.07
**Comparisons between ethnoracially minoritized and White youth**											
Measurement equivalence											
Configural (unconstrained)	6158.70	5984.06			−2898.73	55	1.23				
Structural (equal means)	6088.92	5952.39	−69.78	−31.67	−2903.25	43	1.22	1.25	7.25	12	.84
Dispersion (equal means and variances)	6016.37	5917.94	−72.55	−34.45	−2906.38	31	1.19	1.32	4.76	12	.97
Distributional (equal means, variances, probabilities)	6002.14	5913.24	−14.23	−4.70	−2909.12	28	1.24	0.75	7.31	3	.06
Predictive similarity											
Freely estimated	11561.58	11425.05			−5639.97	43	1.03				
Equally estimated	11480.06	11391.15	−81.53	−33.90	−5648.33	28	1.04	0.99	16.88	15	.33
Explanatory similarity: Emotional engagement											
Freely estimated	13191.69	13051.98			−6451.75	44	1.07				
Equally estimated	13168.36	13041.35	−23.33	−10.63	−6453.19	40	1.07	1.04	2.76	4	.60
Explanatory similarity: Behavioral engagement											
Freely estimated	12932.72	12793.01			−6322.27	44	1.01				
Equally estimated	12912.33	12785.32	−20.39	−7.69	−6325.17	40	1.00	1.10	5.28	4	.26
Explanatory similarity: Academic aspirations											
Freely estimated	14065.87	13926.17			−6888.85	44	1.08				
Equally estimated	14049.98	13922.97	−15.90	−3.20	−6894.00	40	1.03	1.62	6.36	4	.17
Explanatory similarity: Academic expectations											
Freely estimated	14141.47	14001.77			−6926.65	44	1.03				
Equally estimated	14136.52	14009.51	−4.96	7.75	−6937.27	40	0.97	**1.63**	**13.07**	**4**	**.01**

The predictive and explanatory similarity models testing equivalence between grades included all covariates except grade. The predictive and explanatory similarity models testing equivalence between ethnic-racial backgrounds included all covariates except ethnicity-race. Estimates in boldface indicate whether a freely estimated model is better fitting than an equally estimated model (*p* < .05)

*BIC* Bayesian Information Criterion, *aBIC* sample-size adjusted BIC, *LL* log likelihood, *#fp* number of free parameters, *MLR cf* MLR scaling correction factor, *cd* scaling correction factor for the difference

Subsequent analyses examined the associations between youth background characteristics and the identified school support profiles. There were no substantial changes in profile composition with the use of FIML. There was no evidence for covariate predictive differences between grades or ethnic-racial background based on multiple group analyses (Table 3); when covariate predictors were equally estimated between grades or ethnic-racial background, the covariates similarly predicted profile membership. Thus, associations between youth background covariates and the identified school support profiles are interpreted based on an overall model (Table 4). Youth with higher family financial resources were *less* likely to be in the *moderate general, moderate cultural, & devoid critical support* (#2) profile than the *high general, moderate cultural & critical support* (#3) profile. Youth with higher family support were also *less* likely to be in the *low general, devoid cultural & critical* (#1) and *moderate general, moderate cultural, & devoid critical* (#2) support profiles than the *high general, moderate cultural & critical* (#3) support profile. Compared to sixth graders, ninth graders were *more* likely to be in the *low general, devoid cultural & critical support* (#1) profile than the *moderate general, moderate cultural, & devoid critical* (#2) and *high general, moderate cultural & critical* (#3) support profiles, and *more* likely to be in the *moderate general, moderate cultural, & devoid critical support* (#2) profile than the *high general, moderate cultural & critical support* (#3) profile. Girls were *more* likely than boys to be in the *moderate general, moderate cultural, & devoid critical support* (#2) profile than the *high general, moderate cultural & critical support* (#3) profile and more likely to be in the *high general, moderate cultural & critical support* (#3) profile than the *high general, cultural, & critical support* (#4) profile. Youth with ethnoracially minoritized backgrounds were *more* likely than White youth to be in the *low general, devoid cultural & critical support* (#1) and *moderate general, moderate cultural, & devoid critical support* (#2) profiles than *high general, cultural, & critical support* (#4) profile.

**Table 4:** Multinomial logistic regression analyses: associations between covariates and the 4-profile LPA solution of culturally relevant school support (*n* = 697)

	Profiles 1 VS. 2			Profiles 1 VS. 3			Profiles 1 VS. 4		
	Coef.	SE	OR	Coef.	SE	OR	Coef.	SE	OR
Family SES^a^	0.328	0.308	1.388	0.007	0.286	1.007	−0.084	0.342	0.919
Family social support	−0.029	0.096	0.971	−0.254*	**0.101**	**0.776**	0.246	0.430	1.279
GPA^b^	−0.195	0.169	0.823	−0.190	0.167	0.827	−0.533	0.345	0.587
Ninth grade^c^	0.929*	**0.474**	**2.532**	1.510***	**0.457**	**4.527**	−0.190	0.228	0.827
Girl^d^	−0.383	0.348	0.682	0.119	0.331	1.126	0.519	0.489	1.680
Ethnoracially minoritized^e^	0.256	0.440	1.292	−0.202	0.416	0.817	2.004***	**0.533**	**7.419**
	Profiles 2 VS. 3			Profiles 2 VS. 4			Profiles 3 VS. 4		
	Coef.	SE	OR	Coef.	SE	OR	Coef.	SE	OR
Family SES^a^	−0.321*	**0.154**	**0.725**	−0.413^**†**^	0.229	0.662	−0.092	0.220	0.912
Family social support	−0.224**	**0.078**	**0.799**	0.629^**†**^	0.343	1.876	0.128	0.328	1.137
GPA^b^	0.005	0.128	1.005	−0.504	0.337	0.604	−0.279	0.354	0.757
Ninth grade^c^	0.581*	**0.248**	**1.788**	0.004	0.195	1.004	0.000	0.191	1.000
Girl^d^	0.501*	**0.231**	**1.650**	0.263	0.356	1.301	0.721*	**0.341**	**2.056**
Ethnoracially minoritized^e^	−0.458^**†**^	0.272	0.633	1.075**	**0.355**	**2.930**	0.494	0.336	1.639

Boldface represents significant estimates (*p* < .05) indicating that a given covariate indicator was a significant predictor of profile membership across compared profiles; estimates reflect the effects of the predictors on the likelihood of membership into the first versus second listed reference profile. Profile 1: Low general, devoid cultural & critical support; Profile 2: Moderate general, moderate cultural, & devoid critical support; Profile 3: High general, moderate cultural & critical support; and Profile 4: High general, cultural, & critical support

*VS.* versus, *Coef.* coefficient, *SE* standard error, *OR* odds ratio, *SES* socioeconomic status, *GPA* grade-point average assessed in the fall semester of 2019

^a^SES = socioeconomic status

^b^Grade point average assessed in the fall semester of 2019

^c^Ninth grade (1 = *ninth grade*; 0 = *sixth grade*)

^d^Girl (1 = *girl*; 0 = *boy*)

^e^Ethnoracially minoritized (1 = *ethnoracially minoritized*; 0 = *White*)

^†^*p* < .10; *p < .05; **p < .01; ***p < .001

### Associations Between School Support Profiles and Youth Academic Functioning

Aim 2 examined how the school support profiles identified in Aim 1 were related to markers of academic functioning (Table 5) while accounting for the covariates. There were no substantial changes in profile composition with the use of FIML. Supporting the study’s hypotheses, pairwise z-tests revealed that emotional and behavioral engagement scores were significantly higher for those in the *high general, cultural, & critical support* profile (#4) compared to all other profiles. Emotional and behavioral engagement was also higher in the *high general, moderate cultural & critical support* (#3) and *moderate culturally and critically devoid* (#2) profiles compared to the *high culturally and critically devoid support* profile (#1). There were no significant differences across profiles for academic aspirations or expectations, with one exception for academic expectations described in the next section.

**Table 5:** Aim 2. Associations between culturally relevant school support latent profile solution and adolescent academic functioning indicators

	1. Behavioral Engagement^a^ (*n* = 697)		2. Emotional Engagement^a^ (*n* = 697)		3. Academic Aspirations^a^ (*n* = 697)		4. Academic Expectations^b^ (*n* = 699)			
							*Ethnoracially minoritized youth*		*White youth*	
Academic Functioning Scores by Profile	*M*	*SE*	*M*	*SE*	*M*	*SE*	*M*	*SE*	*M*	*SE*
1. Low general, devoid cultural & critical support	2.65	0.14	1.82	0.16	4.93	0.26	4.63	0.35	3.42	0.60
2. Moderate general, moderate cultural, & devoid critical support	2.74	0.06	2.06	0.09	5.17	0.15	4.39	0.15	4.41	0.28
3. High general, moderate cultural & critical support	3.03	0.05	2.56	0.06	5.23	0.12	4.33	0.12	5.22	0.27
4. High general, cultural, & critical support	3.38	0.06	3.18	0.11	5.00	0.23	4.40	0.29	4.97	0.50
Pairwise Comparisons Across Profiles^c^										
4. High general, cultural, & critical support vs.:										
1. Low general, devoid cultural & critical support	5.05***		5.20***		−0.23		−0.24		1.55*	
2. Moderate general, moderate cultural, & devoid critical support	5.04***		7.34***		0.07		0.01		0.56	
3. High general, moderate cultural & critical support	7.75***		8.37***		−0.17		0.07		−0.25	
3. High general, moderate cultural & critical support vs.:										
1. Low general, devoid cultural & critical support	−3.93***		−5.12***		−0.06		−0.30		1.80**	
2. Moderate general, moderate cultural, & devoid critical support	0.60		1.50		0.24		−0.06		0.81^**†**^	
2. Moderate general, moderate cultural, & devoid critical support vs.:										
1. Low general, devoid cultural & critical support	−2.81**		−4.79***		−0.30		−0.24		0.99	

*M* mean, *SE* standard error

^a^Findings in Columns 1, 2, and 3 are based on three separate models

^b^Findings in Column 4 for academic expectations across profiles are based on a multiple group analysis comparing ethnoracially minoritized youth and White youth. Table 3 includes multiple group comparison tests indicating that there were only significant explanatory differences across ethnicity-race for academic expectations

^c^Estimates in boldface represent significant *z*-tests (*p* < .05) for mean level differences across profiles based on pairwise comparisons, with the following covariates included and assessed between December 2019 and January 2020: perceived family socioeconomic status, family social support, girl, a dummy-coded ethnicity-race variable (for Models 1–3), and grade

^**†**^*p* < .10; *p < .05; **p < .01; ***p < .001

### Developmental and Ethnoracial Differences

The preliminary prediction based on exploratory Aim 3 that the hypothesized profiles would be most strongly associated with the academic functioning of ethnoracially minoritized youth (compared to White youth) and youth in 9^th^ grade (compared to 6^th^ grade) was not supported (see explanatory similarity tests in Table 3). There were no grade differences in academic functioning measures across profiles. One explanatory similarity difference was evident when examining academic expectations. For White youth, those in the *high general, cultural, & critical support* (#4) and *high general, moderate cultural & critical support* (#3) profiles had higher academic expectations than those in the *low general, devoid cultural & critical support* (#1) profile. For ethnic-racial minoritized youth, there were no significant differences in academic expectations across profiles.

## Discussion

In a rapidly diversifying world, increased scrutiny and threats against efforts to incorporate multicultural education in schools, ethnic studies, and mere conversations about race and ethnicity exist across the United States (Koyama, 2024). Yet, youth’s developmental needs for relatedness emphasize the importance of authentic school connections that validate and address the realities of living in multicultural societies and affirm adolescents as cultural beings (García Coll et al., 1996; Huguley et al., 2019). This study took a person-centered approach to identify adolescent-reported profiles of culturally relevant school support and tested their associations with measures of academic functioning, while accounting for youth background characteristics. The study also explored potential developmental and ethnic-racial differences across the associations between culturally relevant school support profiles and academic functioning.

Aligned with theory on culturally relevant school support practices (Byrd, 2016; Ladson-Billings, 1995, 2004), this study characterized students’ distinct collective experiences of general, cultural, and critical support in school to identify four culturally relevant school support profiles that adolescents reported experiencing (Aim 1). Mostly consistent with the expectations, these profiles included: (a) *high general, cultural, & critical support* characterized by relatively high general, cultural, & critical support (#4) compared to all other profiles, (b) *high general, moderate cultural & critical support* (#3), (c) *moderate general, moderate cultural, & devoid critical support* (#2), and (d) *low general, devoid cultural & critical support* (#1) profiles with relatively low levels of teacher support and very low (devoid) promotion of cultural competence and critical consciousness socialization. Further, while accounting for key background characteristics, youth in the *high general, cultural, & critical support* profile (#4) had consistently higher concurrent emotional and behavioral engagement than other profiles (Aim 2). Youth in the *high general, moderate cultural & critical support* (#3) and *moderate general, moderate cultural, & devoid critical support* (#2) profiles showed higher emotional and behavioral engagement than the *low general, devoid cultural & critical support* (#1) profile. The study’s exploratory aim sought to examine developmental age and ethnoracial group membership differences in the linkages between school support profiles and academic outcomes. No significant differences were found between early (6^th^ grade) and middle adolescent (9^th^ grade) youth, pointing to the relevance and associations of the identified profiles across development. When comparing White and ethnoracially minoritized youth, it was found that among White youth, those in the *high general, cultural, & critical support* and *high general, moderate cultural & critical support* profiles had higher academic expectations than those in the *low general, devoid cultural & critical support* profile. Taken together, the present study extends developmental scholarship of culturally relevant support in schools by describing adolescent-centered accounts of their teachers’ support through general support and learning about other cultures and social inequities, documenting that a higher degree of culturally relevant school support (i.e., high general, cultural, and critical support) is generally associated with greater academic functioning.

### Identifying Profiles of Culturally Relevant Support in School (Aim 1)

Prior work emphasizes the importance of culturally relevant support for youth development, but youth encounter varying degrees of this type of support in school (Byrd, 2016; Ladson-Billings, 1995, 2004). Mostly aligned with the study’s hypotheses (Aim 1), four profiles were identified: *low general, devoid cultural & critical support* (#1), *moderate general, moderate cultural, & devoid critical support* (#2), *high general, moderate cultural & critical support* (#3), and *high general, cultural, & critical support* (#4). Contrary to expectations, a profile with relatively similar and low levels of support across dimensions was not identified. Instead, all four profiles had higher general support than cultural and critical support (at varying degrees) and either higher promotion of cultural competence than critical consciousness socialization (for Profiles 1 and 2, at varying degrees) or similar promotion of cultural competence and critical consciousness socialization support levels (for Profiles 3 and 4). The critically devoid profiles (with low to moderate general and cultural support; Profiles #1 and #2) had relatively lower but discrepantly higher promotion of cultural competence than critical consciousness socialization, potentially because this might be more aligned with mainstream educational values that do not address or integrate critical perspectives on social inequities (Ladson-Billings, 1995). Most adolescents reported that their schools provided *high general, moderate cultural & critical support* (Profile 3; 49%), followed by *moderate general, moderate cultural, & devoid critical support* (Profile 2; 34%). A small share of adolescents (17%) was more likely in the *low general, devoid cultural & critical* (#1) or *high general, cultural, & critical support* (#4) profiles, representing the lowest or highest cultural and critical support levels, respectively, in the study sample. The findings highlight that, although youth perceive relatively higher levels of general than cultural or critical support, there is a significant degree of difference across school support dimensions within and across profiles, replicating past findings on variability in school ethnic-racial socialization (Byrd & Ahn, 2020).

Although not a central question to the current study, potential background characteristics that might predict the support adolescents receive and perceive in school were tested. Girls were *more* likely than boys to be in a profile characterized by relatively devoid critical support (*moderate general, moderate cultural, & devoid critical support* [#2]) and *less* likely to be in the *high general, cultural, & critical support* (#4) profile versus a *high general, moderate cultural & critical support* [#3] profile. These findings are inconsistent with some research suggesting that girls are more likely than boys to receive higher teacher support (Rueger et al., 2010) and more cultural socialization, at least in the family context (Huynh & Fuligni, 2008). Interestingly, those with a higher family SES or social support were more likely to be in the *high general, moderate cultural & critical support* (#3) profile compared to the *moderate general, moderate cultural, & devoid critical support* (#2) profile, suggesting that family-level factors might help support the likelihood of experiencing at least moderate critical support, perhaps from the support youth receive in out-of-school contexts given family-level resources. Future research might examine the conditions under which this association emerges and the school and community conditions contributing to the likelihood of experiencing culturally relevant support in schools. For instance, teachers might be less likely to provide culturally relevant support if they feel unprepared to engage in culturally relevant practices with students in multicultural classrooms (Chahar Mahali & Sevigny, 2022).

### Profiles of Culturally Relevant Support in School and Academic Functioning (Aim 2)

Aligned with theory on culturally relevant pedagogy (Ladson-Billings, 1995) and proximal school ethnic-racial socialization processes (Saleem & Byrd, 2021), this study examined the role of school support profiles in promoting various aspects of academic functioning. Supporting the study’s hypotheses, youth in profiles with relatively higher culturally relevant support also had higher levels of emotional and behavioral academic engagement compared to youth in profiles with relatively low support in school; the lowest engagement scores were evident in the *low general, devoid cultural & critical* (#1) profile. These findings accounted for prior academic achievement (based on GPA), which provided a robust test of the associations. Because research shows declines in school engagement across adolescence (Wang & Eccles, 2012), these findings emphasize the importance of leveraging culturally relevant practices in schools to maximize student engagement. These findings also align with research on the academic benefits of ethnic studies curriculum for youth (Dee & Penner, 2017), highlighting the importance of general, cultural, and critical support experiences that meet youth needs of relatedness and engaging in a multicultural world.

Interestingly, whereas teacher support was positively correlated with students’ academic aspirations and expectations at a bivariate level, the documented profiles were not significantly associated with these academic measures (see exploratory Aim 3 for one exception). It is plausible that youth academic aspirations and expectations are better informed by youths’ assessment of their academic standing and financial resources rather than teacher support. For instance, GPA and family socioeconomic status were correlated with students’ academic aspirations and expectations. This finding is consistent with prior research on the links between parent educational attainment and youth’s educational aspirations and expectations (Lui et al., 2014). Future work needs to examine how holistic school support and related interventions might disrupt strong intergenerational linkages between family socioeconomic status and youth aspirations for higher education. One potential route through which culturally relevant school practices can promote academic expectations, aspirations, and resources is via the promotion of intergroup connections among students of different socioeconomic class, race, and ethnic backgrounds that have been shown to boost academic achievement among ethnically and racially diverse youth (Lessard & Juvonen, 2019). Further, more research is needed to identify how school and teacher culturally relevant practices can inform and foster navigational capital (i.e., skills needed to navigate educational and other institutions that could be exclusionary or hostile to youth of color; Yosso, 2005) that is distributed across co-ethnic connections and community members and is theorized to promote academic aspirations and expectations in culturally authentic ways.

### Developmental and Ethnoracial Differences (Exploratory Aim 3)

Contrary to the study’s preliminary hypothesis on the relative strength of associations between culturally relevant school support profiles and academic functioning across grades, no differences emerged. Additional factors not tested in this study, such as how youth cope with daily ethnic-racial tensions in school across development (Hughes & Watford, 2021), could be the basis for testing possible developmental differences over time. Future research should continue to examine possible developmental differences in the experiences and possible outcomes of culturally relevant school support.

Only one ethnoracial difference emerged for academic expectations, and it was inconsistent with the study’s preliminary hypothesis on ethnoracial differences but consistent with the overall hypothesis on the role of culturally relevant school support profiles on academic functioning. White youth in the *high general, cultural, & critical support* (#4) and *high general, moderate cultural & critical support* (#3) profiles had higher academic expectations than White youth in the *low general, devoid cultural & critical support* (#1) profile; no associations were significant for ethnic-racial minoritized youth. This finding suggests that experiencing high general and at least moderate cultural and critical support might provide White youth in the study sample with the competencies and self-efficacy necessary to interact with a diverse student body and engage with the complexities of ethnic-racial and social relations (Wantchekon & Umaña-Taylor, 2024); these experiences, in turn, could promote their goals to pursue further educational milestones. This finding accounted for the role of key background covariates, including family SES and social support, emphasizing the benefits of experiencing relatively high culturally relevant school support across domains for White youth’s academic adjustment. It is possible that culturally relevant school support, the way it was assessed in this study, might not be sufficient to compensate for the daily lived ethnic-racial tensions that ethnic-racial minoritized youth encounter in school (Hughes & Watford, 2021) or disrupt the close link between parent educational attainment and youth’s educational expectations (Lui et al., 2014) that could hamper academic pursuits. That is, additional supports are likely needed to promote academic aspirations and expectations among ethnoracially minoritized youth typically underrepresented in higher education. Recent findings suggest that critical consciousness socialization in school was associated with increased ethnic-racial identity exploration among White youth (Kornienko et al., 2024), which might further promote their academic aspirations.

Of note, there were no significant differences between Profiles #2 and #3 for most academic functioning indicators; academic expectations were higher, at a marginally significant trend (*p* < .10), for those in Profile #3 (*high general, moderate cultural & critical support*) compared to those in Profile #2 (*moderate general, moderate cultural, & devoid critical support*) for White youth. These two profiles represented most youth in the sample, and when comparing Profile 2 (*moderate general, moderate cultural, & devoid critical support*) and Profile 3 (*high general, moderate cultural & critical support*), most of the background characteristics (except GPA) predicted differences in profile membership. Overall, these findings suggest there are nuanced background characteristic differences, likely embedded in larger systemic and societal influences, between the most common profiles that might develop and increase in significance over time.

### Developmental and Applied Implications

Whereas the bulk of research has revealed how family ethnic-racial socialization and ERI development are promotive and protective factors for academic outcomes (Miller-Cotto & Byrnes, 2016; Umaña-Taylor, 2016), the present study extends this literature by documenting that some adolescent academic outcomes are better facilitated when their teachers show higher levels of culturally relevant support – that is, overall support and help with learning about both social justice issues and diverse cultures. These notions of culturally affirming school settings represent promotive proximal school contexts long conceptualized by integrative models of minority youth development (García Coll et al., 1996) and ethnic-racial socialization (Huguley et al., 2019). Accordingly, the present study advances a youth-centered account of experiences with opportunities to learn and navigate an increasingly multicultural yet socially stratified and inequitable world. Using a person-centered approach enabled the present study to characterize *heterogeneity* (Suzuki et al., 2021) in the extent to which students reported that their schools were supportive, culturally affirming, and critically aware and link such emerging profiles to academic functioning.

This study highlights the importance of students’ cultural competence and critical consciousness support needs beyond experiences of general teacher support. *Most* students received at least some level of cultural or critical support, but some did not, such as those classified into the *low general, devoid cultural & critical* profile (#1). School-wide ethnic-racial inclusivity efforts that integrate culturally relevant practices likely help foster academic functioning in youth (Nishina et al., 2019) by supporting a greater sense of school belonging (Vang & Nishina, 2022). Emerging meta-analytic evidence reveals that multiculturalism school climate promotes intergroup attitudes, academic achievement, motivation, and belonging, whereas critical consciousness school climates enhance academic achievement, motivation, engagement, social belonging, and well-being (Bardach et al., under review), highlighting the need for ethnic-racial inclusivity efforts to integrate support for students’ cultural competence and critical consciousness, and, as the present study’s findings suggest, general teacher support.

One way to integrate culturally relevant, sensitive, and responsive practices is through implementing youth-centered and social identity-affirming universal intervention programs (e.g., Hoffman & Umaña‐Taylor, 2023). Specifically, emerging evidence on the benefits of ethnic-racial identity development interventions, an example of culturally relevant practices (Umaña-Taylor, in press), for youth ethnic-racial identity and academic well-being point to the overall advantages of encouraging identity-related exploration for both ethnoracially minoritized and White youth (Umaña-Taylor et al., 2018; Wantchekon & Umaña-Taylor, 2024). Another way to integrate culturally responsive practices into schools is by adopting an ethnic studies curriculum. Recent evidence from quasi-experimental (Bonilla et al., 2021) and experimental (Gillespie et al., 2024) research designs on the effects of ethnic studies’ curriculum (i.e., promotion of cultural socialization, cultural competence, and critical reflection) on adolescent academic and psychological adjustment outcomes revealed positive effects for both ethnoracially minoritized and White youth (Gillespie et al., 2024).

Taken together, the present results and the above-noted effects of ERI development interventions and ethnic curriculum programs point to the benefits of culturally responsive school practices for adolescent academic and developmental outcomes. This pattern of findings is informative in the current contested climate across the U.S., whereby some states are restricting, whereas other states are expanding their educational policies on bias, racism, and history of specific ethnoracial groups (see Stout & Wilburn, 2022, for the current map). Opponents of discussing race or ethnicity in the classroom argue that it promotes guilt, discomfort, and negative affect among White youth (McGee et al., 2021, September 27). Recent findings suggest otherwise, such that school critical consciousness socialization was unrelated to feeling negative about one’s ethnicity or race but was positively associated with increased exploration of the meaning of one’s ethnicity and race among White youth from the Southwestern U.S. (Kornienko et al., 2024). Furthermore, the present cross-sectional findings and other experimental and quasi-experimental evidence point to psychosocial and academic benefits for all and specifically White youth (e.g., Bonilla et al., 2021; Gillespie et al., 2024; Umaña-Taylor et al., 2018). Thus, more basic research and translational efforts are needed to probe how culturally relevant practices can be integrated across schools to support educators and communities and provide developmental benefits for youth who grow up in diverse, multicultural, and pluralistic societies.

### Limitations and Future Directions

The present study benefited from several key strengths, including a large and ethnoracially diverse sample of early and middle adolescents, multi-method assessment using adolescent self-report and school records (e.g., GPA), and comprehensive measurement of theoretically aligned aspects of cultural and critical support practices. Despite these strengths, a few noteworthy limitations represent directions for future research. First, due to COVID-19 pandemic-related disruptions in data collection, this study uses cross-sectional and primarily self-reported data from adolescents collected right before the pandemic (winter 2019–2020), complemented by objective school records on GPA as a covariate (fall 2019). Given the cross-sectional nature of the data, potential bidirectional associations between academic functioning and culturally relevant school support profiles over time were not tested. Early GPA did not predict differences across profiles, though other measures of academic functioning (such as academic engagement) might also predict culturally relevant school support profiles. However, prior GPA was included as a covariate to account for the possible role of early academic functioning in predicting the likelihood of students being more motivated to seek multiple types of support from teachers.

The present study focused on characterizing culturally relevant school support by integrating general, cultural, and critical support, as advocated by recent calls in the field (e.g., Saleem & Byrd, 2021). Future work can incorporate additional culturally relevant support dimensions (e.g., high teacher expectations) and whether youth are more likely to experience culturally relevant support in specific courses (e.g., social studies vs. math). Moving beyond the within-setting cultural socialization processes, future work needs to examine the role of family and community-based culturally responsive and ethnic-racial socialization processes that can shape and boost how schools can support students (Byrd & Ahn, 2020; Hernández et al., 2023). Importantly, school and family ethnic-racial socialization processes need to be considered in tandem to ascertain the directionality and unique contribution of these socializing influences. For instance, Latinx immigrant parents tend to endorse socializing their children to understand the value of diversity and cultural differences (Ayón, 2018). These values might also lead to parental encouragement and modeling that will enhance youth seeking out relevant opportunities in school to engage with and receive cultural or critical support from teachers and staff. Thus, future work needs to better understand the extent to which adolescents from ethnoracially minoritized backgrounds may be more likely to seek out culturally relevant experiences in school that are consistent with the messages they receive in their families and communities.

Understanding the alignment between messages, practices, and expectations regarding cultural support between schools and parents is vital for promoting family engagement in school and school-community offerings of a culturally engaging curriculum. Efforts to support multicultural education are often born from community and student demands of and resistance to a curriculum that does not reflect students’ communities. Future research needs to investigate how community and student efforts enhance culturally relevant support in school and community spaces, which ultimately supports academic success and competence in multicultural environments for all students (Cabrera et al., 2013).

Currently, efforts to implement and evaluate culturally responsive teaching practices are met with increased challenges, scrutiny, and threats due to a socio-political climate that is channeled through parental concerns, school district guidelines, and state-wide policies (Koyama, 2024; Umaña-Taylor, in press). Further, according to the Pew Research Center (2023), a nationwide comparative analysis of school district mission statements’ content reveals that 34% of school districts underscore the importance of equity, diversity, and inclusion and that the political leaning of the school district location (i.e., vote share in 2020) divides whether this topic is noted as important. Specifically, the importance of diversity, equity, and inclusion is highlighted within 56% of mission statements from school districts in Democratic-leaning areas as compared to 26% in Republican-leaning areas, pointing to inequities in school provisions of culturally affirming experiences that address youth’s developmental needs for ethnic-racial identity development, relatedness, authentic school connection, academic success, health, and well-being.

Another fruitful avenue for future work entails examining the benefits of culturally relevant support that may extend beyond academic functioning, including ethnic-racial identity development (Umaña-Taylor, 2023) and better psychological adjustment (Byrd & Ahn, 2020). More attention is also needed to test the mechanisms through which culturally relevant school support practices promote academic functioning, with candidate processes of school belonging (e.g., Tan, 2001; Vang & Nishina, 2022). A person-centered analysis of culturally relevant school support practices will help extend research on addressing the holistic needs of youth to promote their well-being.

## Conclusion

Adolescents need authentic culturally relevant school support that validates the realities of living in multicultural societies. Research linking general teacher support to adolescent academic functioning has rarely also considered the cultural competence and critical consciousness support students receive in school. The present study extends research by estimating how person-centered profiles of culturally relevant school support, which include teacher support, promotion of cultural competence, and critical consciousness socialization, relate to adolescent academic functioning. Findings from the current study highlight that adolescents from multiple ethnoracial backgrounds are exposed to varying degrees of culturally relevant support in their schools. Adolescents experiencing higher levels of *culturally relevant support* demonstrated higher behavioral and emotional academic engagement levels compared to all other profiles. Study findings highlight that providing general, cultural competence, and critical consciousness support in school is promising for improving youth’s academic functioning at a critical time when declines are typically observed.

## Appendix

Table 6

**Table 6:** Scale items used for the latent profiles

**Promotion of Cultural Competence Scale Items**
1. Your classes teach you about diverse cultures and traditions.
2. You have learned about new cultures and traditions at school.
3. You have the chance to learn about the cultures of others.
4. In school you get to do things that help you learn about people of different races and cultures.
5. At your school, they encourage you to learn about different cultures.
**Critical Consciousness Socialization Scale Items**
1. Your teachers encourage awareness of social issues affecting your culture.
2. Teachers teach about racial inequality in the United States.
3. In your classes you have learned about how race/ethnicity plays a role in who is successful.
4. You have opportunities to learn about social justice.
**Teacher Support Scale Items**
1. Our teachers treat us fairly.
2. When I need extra help, I can get it.
3. My teachers are interested in me as a person.
4. Our teachers are nice and friendly.

Citation for *Promotion of Cultural Competence* and *Critical Consciousness Socialization* Scales: Byrd, C. M. (2017). The complexity of school racial climate: Reliability and validity of a new measure for secondary students. *British Journal of Educational Psychology*, *87*(4), 700–721. 10.1111/bjep.12179. Citation for *Teacher Support* Scale: Torsheim, T., Wold, B., & Samdal, O. (2000). The teacher and classmate support scale: factor structure, test-retest reliability and validity in samples of 13-and 15-year-old adolescents. *School Psychology International*, *21*(2), 195–212. 10.1177/0143034300212006

## References

[CR1] Allen, K., Kern, M. L., Vella-Brodrick, D., Hattie, J., & Waters, L. (2016). What schools need to know about fostering school belonging: a meta-analysis. *Educational Psychology Review*, *30*(1), 1–34. 10.1007/s10648-016-9389-8.

[CR2] Asparouhov, T., & Muthén, B. (2014). Auxiliary variables in mixture modeling: Three-step approaches using Mplus. *Structural Equation Modeling: A Multidisciplinary Journal*, *21*(3), 329–341. 10.1080/10705511.2014.915181.

[CR3] Ayón, C. (2018). Latino immigrant family socialization scale: development and validation of a multidimensional ethnic-racial socialization measurement. *Social Work*, *63*(3), 222–233. 10.1093/sw/swy016. 29701823 10.1093/sw/swy016

[CR4] Bakk, Z., & Vermunt, J. K. (2015). Robustness of stepwise latent class modeling with continuous distal outcomes. *Structural Equation Modeling: A Multidisciplinary Journal*, *23*(1), 20–31. 10.1080/10705511.2014.955104.

[CR5] Banfield, J. D., & Raftery, A. E. (1993). Model-based Gaussian and non-Gaussian clustering. *Biometrics*, *49*(3), 803–821. 10.2307/2532201.

[CR6] Bardach, L., Röhl, S., Oczlon, S., Schumacher, A., Lüftenegger, M., Lavelle-Hill, R., Schwarzenthal, M., & Zitzmann, S. Cultural diversity climate at school: a meta-analysis of relationships with intergroup, academic, and socioemotional outcomes. 10.31219/osf.io/fvjks. 10.1037/bul0000454 39652446

[CR7] Barrett, M. (2018). How schools can promote the intercultural competence of young people. *European Psychologist*, *23*, 93–104. 10.1027/1016-9040/a000308.

[CR8] Bonilla, S., Dee, T. S., & Penner, E. K. (2021). Ethnic studies increases longer-run academic engagement and attainment. *Proceedings of the National Academy of Sciences*, *118*(37), e2026386118 10.1073/pnas.2026386118. 10.1073/pnas.2026386118 PMC8449416 34493663

[CR9] Bozdogan, H. (1987). Model selection and Akaike’s information criterion (AIC): The general theory and its analytical extensions. *Psychometrika*, *52*, 345–370. 10.1007/BF02294361.

[CR10] Byrd, C. M. (2015). The associations of intergroup interactions and school racial socialization with academic motivation. *The Journal of Educational Research*, *108*(1), 10–21. 10.1080/00220671.2013.831803.

[CR11] Byrd, C. M. (2016). Does culturally relevant teaching work? An examination from student perspectives. *SAGE Open*, 6(3). 10.1177/2158244016660744.

[CR12] Byrd, C. M. (2017). The complexity of school racial climate: reliability and validity of a new measure for secondary students. *British Journal of Educational Psychology*, *87*(4), 700–721. 10.1111/bjep.12179. 28850714 10.1111/bjep.12179

[CR13] Byrd, C. M., & Ahn, L. H. (2020). Profiles of ethnic-racial socialization from family, school, neighborhood, and the Internet: Relations to adolescent outcomes. *Journal of Community Psychology*, *48*(6), 1942–1963. 10.1002/jcop.22393. 32526066 10.1002/jcop.22393

[CR14] Byrd, C. M., & Chavous, T. (2011). Racial identity, school racial climate, and school intrinsic motivation among African American youth: the importance of person-context congruence. *Journal of Research on Adolescence*, *21*, 849–860. 10.1111/j.1532-7795.2011.00743.x.

[CR15] Byrd, C. M., & Hope, E. C. (2020). Black students’ perceptions of school ethnic-racial socialization practices in a predominantly Black school. *Journal of Adolescent Research*, *35*, 728–753. 10.1177/0743558419897386.

[CR16] Cabrera, N. L., Meza, E. L., Romero, A. J., & Cintli Rodríguez, R. (2013). If there is no struggle, there is no progress”: transformative youth activism and the school of ethnic studies. *The Urban Review*, *45*(1), 7–22. 10.1007/s11256-012-0220-7.

[CR17] Castro-Schilo, L., Ferrer, E., Hernández, M. M., & Conger, R. D. (2016). Developmental consequences of school attachment among students of Mexican origin. *Journal of Research on Adolescence*, *26*(4), 753–768. 10.1111/jora.12223. 28453214 10.1111/jora.12223

[CR18] Chahar Mahali, S., & Sevigny, P. R. (2022). Multicultural classrooms: culturally responsive teaching self-efficacy among a sample of Canadian preservice teachers. *Education and Urban Society*, *54*(8), 946–968. 10.1177/0013124521106.

[CR19] Chang, J., & Le, T. N. (2010). Multiculturalism as a dimension of school climate: the impact on the academic achievement of Asian American and Hispanic youth. *Cultural Diversity & Ethnic Minority Psychology*, *16*(4), 485–492. 10.1037/a0020654. 21058811 10.1037/a0020654

[CR20] Deci, E. L., & Ryan, R. M. (2000). The “what” and “why” of goal pursuits: human needs and the self-determination of behavior. *Psychological Inquiry*, *11*(4), 227–268. 10.1207/s15327965pli1104_01.

[CR21] Dee, T. S., & Penner, E. K. (2017). The causal effects of cultural relevance: evidence from an ethnic studies curriculum. *American Educational Research Journal*, *54*(1), 127–166. 10.3102/0002831216677002.

[CR22] Del Toro, J., & Wang, M. T. (2021). Longitudinal inter-relations between school cultural socialization and school engagement among urban early adolescents. *Journal of Youth and Adolescence*, *50*, 978–991. 10.1007/s10964-020-01377-w. 33442773 10.1007/s10964-020-01377-w

[CR23] Diemer, M. A., Rapa, L. J., Voight, A. M., & McWhirter, E. H. (2016). Critical consciousness: a developmental approach to addressing marginalization and oppression. *Child Development Perspectives*, *10*(1), 216–221. 10.1111/cdep.12193.

[CR24] Ferguson, S. L. G., Moore, E. W., & Hull, D. M. (2020). Finding latent groups in observed data: a primer on latent profile analysis in Mplus for applied researchers. *International Journal of Behavioral Development*, *44*(5), 458–468. 10.1177/0165025419881721.

[CR25] Fernández-Lasarte, O., Goñi, E., Camino, I., & Ramos-Díaz, E. (2019). Apoyo social percibido e implicación escolar del alumnado de educación secundaria—Perceived social support and school engagement in secondary students. *Revista Española de Pedagogía*, *77*(272), 123–142. https://www.jstor.org/stable/26633285.

[CR26] Gale, A. (2020). Examining Black adolescents’ perceptions of in-school racial discrimination: the role of teacher support on academic outcomes. *Children and Youth Services Review*, *116*. 10.1016/j.childyouth.2020.105173.

[CR27] García Coll, C., Crnic, K. A., Lamberty, G., Wasik, B. H., Jenkins, R., Vásquez García, H., & Pipes McAdoo, H. (1996). An integrative model for the study of developmental competencies in minority children. *Child Development*, *67*(5), 1891–1914. 10.2307/1131600. 9022222

[CR28] García Coll, C., & Szalacha, L. A. (2004). The multiple contexts of middle childhood. *The Future of Children*, *14*, 81–96.

[CR29] Gillespie, S., Morency, M. M., Chan, E., & Ferguson, G. M. (2024). Psychological and academic adaptation through universal ethnic studies classes: results of a natural experiment. *Journal of Youth and Adolescence*. 10.1007/s10964-024-02039-x. 10.1007/s10964-024-02039-x 38949674

[CR30] Givens Rolland, R. (2012). Synthesizing the evidence on classroom goal structures in middle and secondary schools. *Review of Educational Research*, *82*(4), 396–435. 10.3102/0034654312464909.

[CR31] Graham, S. (2018). Race/ethnicity and social adjustment of adolescents: How (not if) school diversity matters. *Educational Psychologist*, *53*, 64–77. 10.1080/00461520.2018.1428805.

[CR32] Heberle, A. E., Rapa, L. J., & Farago, F. (2020). Critical consciousness in children and adolescents: a systematic review, critical assessment, and recommendations for future research. *Psychological Bulletin*, *146*(6), 525–551. 10.1037/bul0000230. 32271028 10.1037/bul0000230

[CR33] Hernández, M. M., Robins, R. W., Widaman, K. F., & Conger, R. D. (2016). School belonging, generational status, and socioeconomic effects on Mexican-origin children’s later academic competence and expectations. *Journal of Research on Adolescence*, *26*(2), 241–256. 10.1111/jora.12188. 27231419 10.1111/jora.12188 PMC4876870

[CR34] Hernández, M. M., Safa, M. D., Kornienko, O., Rogers, A. A., & Ha, T. (2023). A person-centered analysis of adolescent multicultural socialization niches and academic functioning. *Journal of Youth and Adolescence*, *52*(11), 2261–2284. 10.1007/s10964-023-01828-0. 37495902 10.1007/s10964-023-01828-0 PMC10495488

[CR35] Hoffman, A. J., & Umaña‐Taylor, A. J. (2023). The promise of leveraging social identities in interventions to enhance the well‐being and lives of adolescents. *Child Development Perspectives*, *17*(3-4), 129–135. 10.1111/cdep.12486.

[CR36] Hughes, D. L., & Watford, J. A. (2021). Racial regularities: Setting-level dynamics as a source of ethnic-racial socialization. *American Journal of Community Psychology*. 10.1002/ajcp.12565. 10.1002/ajcp.12565 34766663

[CR37] Huguley, J. P., Wang, M. T., Vasquez, A. C., & Guo, J. (2019). Parental ethnic-racial socialization practices and the construction of children of color’s ethnic-racial identity: a research synthesis and meta-analysis. *Psychological Bulletin*, *145*(5), 437–458. 10.1037/bul0000187. 30896188 10.1037/bul0000187

[CR38] Huynh, V. W., & Fuligni, A. J. (2008). Ethnic socialization and the academic adjustment of adolescents from Mexican, Chinese, and European backgrounds. *Developmental Psychology*, *44*(4), 1202–1208. 10.1037/0012-1649.44.4.1202. 18605847 10.1037/0012-1649.44.4.1202

[CR39] Kornienko, O., Umaña-Taylor, A. J., Hernández, M. M., & Ha, T. (2024). Friendship network and school socialization correlates of adolescent ethnic-racial identity development. *Journal of Youth and Adolescence*. 10.1007/s10964-024-02052-0. 10.1007/s10964-024-02052-0 PMC11466979 39023840

[CR40] Koyama, J. (2024). The bans on teaching CRT and other ‘divisive concepts’ in America’s public schools. *Journal of Educational Administration and History*, *56*(1), 69–83. 10.1080/00220620.2023.2259813.

[CR41] Ladson-Billings, G. (1995). Toward a theory of culturally relevant pedagogy. *American Educational Research Journal*, *32*(3), 465–491.

[CR42] Ladson-Billings, G. (2004). New directions in multicultural education. In J. A. Banks & C. A. M. Banks (Eds.), *Handbook of research on multicultural education* (Vol. 2, pp. 50–65). Jossey-Bass.

[CR43] Lessard, L. M., & Juvonen, J. (2019). Cross-class friendship and academic achievement in middle school. *Developmental Psychology*, *55*(8), 1666–1679. 10.1037/dev0000755. 31094557 10.1037/dev0000755 PMC6711167

[CR44] Lo, Y., Mendell, N. R., & Rubin, D. B. (2001). Testing the number of components in a normal mixture. *Biometrika*, *88*(3), 767–778. 10.1093/biomet/88.3.767.

[CR45] Lui, C. K., Chung, P. J., Wallace, S. P., & Aneshensel, C. S. (2014). Social status attainment during the transition to adulthood. *Journal of Youth and Adolescence*, *43*(7), 1134–1150. 10.1007/s10964-013-0030-6. 24129883 10.1007/s10964-013-0030-6 PMC3989469

[CR46] Luter, D. G., Mitchell, A. M., & Taylor, H. L. (2017). Critical consciousness and schooling: The impact of the community as a classroom program on academic indicators. *Education Sciences*, *7*(1), 25 10.3390/educsci7010025.

[CR47] Martinez-Fuentes, S., Jager, J., & Umaña-Taylor, A. J. (2021). The mediation process between Latino youths’ family ethnic socialization, ethnic-racial identity, and academic engagement: Moderation by ethnic-racial discrimination? *Cultural Diversity and Ethnic Minority Psychology*, *27*(2), 296–306. 10.1037/cdp0000349. 32406701 10.1037/cdp0000349

[CR48] Masyn, K. E. (2013). Latent class analysis and finite mixture modeling. In T. D. Little (Ed.), *The Oxford handbook of quantitative methods* (Vol. 2, pp. 551–611). Oxford University Press.

[CR49] Mathews, C. J., Medina, M. A., Bañales, J., Pinetta, B. J., Marchand, A. D., Agi, A. C., Miller, S. M., Hoffman, A. J., Diemer, M. A., & Rivas-Drake, D. (2020). Mapping the intersections of adolescents’ ethnic-racial identity and critical consciousness. *Adolescent Research Review*, *5*(4), 363–379. 10.1007/s40894-019-00122-0.

[CR50] McGee, E. O., White, D. T., & Parker, L. (2021, September 27). We taught Critical Race Theory. *Inside Higher Ed*. https://www.insidehighered.com/views/2021/09/28/what-white-students-say-about-critical-race-theory-course-opinion.

[CR51] Meyer, J. P., & Morin, A. J. (2016). A person‐centered approach to commitment research: theory, research, and methodology. *Journal of Organizational Behavior*, *37*(4), 584–612. 10.1002/job.2085.

[CR52] Miller-Cotto, D., & Byrnes, J. P. (2016). Ethnic/racial identity and academic achievement: a meta-analytic review. *Developmental Review*, *41*, 51–70. 10.1016/j.dr.2016.06.003.

[CR53] Morin, A. J. S., Meyer, J. P., Creusier, J., & Biétry, F. (2015). Multiple-group analysis of similarity in latent profile solutions. *Organizational Research Methods*, *19*, 231–254. 10.1177/1094428115621148.

[CR54] Muthén, B. (2004). Latent variable analysis: Growth mixture modeling and related techniques for longitudinal data. In D. S. Kaplan (Ed.), *The SAGE handbook of quantitative methodology for the social sciences* (pp. 346–369). Sage. 10.4135/9781412986311.n19.

[CR55] Nishina, A., Lewis, J. A., Bellmore, A., & Witkow, M. R. (2019). Ethnic diversity and inclusive school environments. *Educational Psychologist*, *54*(4), 306–321. 10.1080/00461520.2019.1633923.

[CR56] Nylund-Gibson, K., & Choi, A. Y. (2018). Ten frequently asked questions about latent class analysis. *Translational Issues in Psychological Science*, *4*(4), 440–461. 10.1037/tps0000176.

[CR57] Nylund-Gibson, K., Grimm, R. P., & Masyn, K. E. (2019). Prediction from latent classes: a demonstration of different approaches to include distal outcomes in mixture models. *Structural Equation Modeling: A Multidisciplinary Journal*, *26*(6), 967–985. 10.1080/10705511.2019.1590146.

[CR58] Odabas, M., & Aragão, C. (2023). *School eistrict mission statements highlight a partisan divide over diversity, equity and inclusion in K-12 education*. https://www.pewresearch.org/social-trends/2023/04/04/school-district-mission-statements-highlight-a-partisan-divide-over-diversity-equity-and-inclusion-in-k-12-education/.

[CR59] Olivera-Aguilar, M., & Rikoon, S. H. (2017). Assessing measurement invariance in multiple-group latent profile analysis. *Structural Equation Modeling: A Multidisciplinary Journal*, *25*(3), 439–452. 10.1080/10705511.2017.1408015.

[CR60] Paizan, M. A., Benbow, A. E. F., & Titzmann, P. F. (2024). Relationship quality in student-teacher-dyads: Comparing student and teacher determinants in multicultural classrooms. *International Journal of Intercultural Relations*, 101. 10.1016/j.ijintrel.2024.102006.

[CR61] Pew Research Center. (2019, April 22). *A changing world: Global views on diversity, gender equality, family life and the importance of religion*. https://www.pewresearch.org/global/2019/04/22/how-people-around-the-world-view-diversity-in-their-countries/.

[CR62] Roorda, D. L., Jak, S., Zee, M., Oort, F. J., Koomen, H. M. Y., & Dowdy, E. (2019). Affective teacher–student relationships and students’ engagement and achievement: A meta-analytic update and test of the mediating role of engagement. *School Psychology Review*, *46*(3), 239–261. 10.17105/spr-2017-0035.V46-3.

[CR63] Rueger, S. Y., Malecki, C. K., & Demaray, M. K. (2010). Relationship between multiple sources of perceived social support and psychological and academic adjustment in early adolescence: Comparisons across gender. *Journal of Youth and Adolescence*, *39*(1), 47–61. 10.1007/s10964-008-9368-6. 20091216 10.1007/s10964-008-9368-6

[CR64] Saleem, F. T., & Byrd, C. M. (2021). Unpacking school ethnic‐racial socialization: a new conceptual model. *Journal of Social Issues*, *77*(4), 1106–1125. 10.1111/josi.12498.

[CR65] Satterthwaite-Freiman, M., & Umaña-Taylor, A. J. (2024). Application of the enduring legacy of the integrative model to investigating white adolescent ethnic-racial identity development. *Human Development*, *68*(3), 121–138. 10.1159/000534965.

[CR66] Schachner, M. K., Schwarzenthal, M., van de Vijver, F. J. R., & Noack, P. (2019). How all students can belong and achieve: effects of the cultural diversity climate amongst students of immigrant and nonimmigrant background in Germany. *Journal of Educational Psychology*, *111*(4), 703–716. 10.1037/edu0000303.

[CR67] Schwarz, G. (1978). Estimating the dimension of a model. *The Annals of Statistics*, *6*, 461–464. 10.1214/aos/1176344136.

[CR68] Sclove, S. L. (1987). Application of model-selection criteria to some problems in multivariate analysis. *Psychometrika*, *52*, 333–343. 10.1007/BF02294360.

[CR69] Seider, S., Clark, S., & Graves, D. (2020). The development of critical consciousness and its relation to academic achievement in adolescents of color. *Child Development*, *91*, e451–e474. 10.1111/cdev.13262. 31140588 10.1111/cdev.13262

[CR70] Seider, S., Graves, D., El-Amin, A., Kelly, L., Soutter, M., Clark, S., Jennett, P., & Tamerat, J. (2023). The development of critical consciousness in adolescents of color attending “opposing” schooling models. *Journal of Adolescent Research*, *38*(1), 3–47. 10.1177/07435584211006466.

[CR71] Skinner, E. A., Kindermann, T. A., & Furrer, C. J. (2009). A motivational perspective on engagement and disaffection: Conceptualization and assessment of children’s behavioral and emotional participation in academic activities in the classroom. *Educational and Psychological Measurement*, *69*(3), 493–525. 10.1177/0013164408323233.

[CR72] Sladek, M. R., Umaña-Taylor, A. J., Hardesty, J. L., Aguilar, G., Bates, D., Bayless, S. D., Gomez, E., Hur, C. K., Ison, A., Jones, S., Luo, H., Satterthwaite-Freiman, M., & Vazquez, M. A. (2022). So, like, it’s all a mix of one”: Intersecting contexts of adolescents’ ethnic-racial socialization. *Child Development*, *93*(5), 1284–1303. 10.1111/cdev.13756. 35366330 10.1111/cdev.13756

[CR73] Stout, C., & Wilburn, T. (2022, February 1). CRT Map: Efforts to restrict teaching racism and bias have multiplied across the US. *Chalkbeat*. https://www.chalkbeat.org/22525983/map-critical-race-theory-legislation-teaching-racism/.

[CR74] Suzuki, S., Morris, S. L., & Johnson, S. K. (2021). Using QuantCrit to advance an anti-racist developmental science: applications to mixture modeling. *Journal of Adolescent Research*, *36*(5), 535–560. 10.1177/07435584211028229.

[CR75] Tan, G. (2001). I want my teachers to like me”: multiculturalism and school dropout rates among Mexican Americans. *Equity & Excellence in Education*, *34*(2), 35–42. 10.1080/1066568010340206.

[CR76] Tao, Y., Meng, Y., Gao, Z., & Yang, X. (2022). Perceived teacher support, student engagement, and academic achievement: a meta-analysis. *Educational Psychology*, *42*, 401–420. 10.1080/01443410.2022.2033168.

[CR77] Tofighi, D., & Enders, C. K. (2008). Identifying the correct number of classes in growth mixture models. In G. R. Hancock & K. M. Samuelsen (Eds.), *Advances in latent variable mixture models* (pp. 317–341). Information Age.

[CR78] Torsheim, T., Wold, B., & Samdal, O. (2000). The teacher and classmate support scale: factor structure, test-retest reliability and validity in samples of 13-and 15-year-old adolescents. *School Psychology International*, *21*(2), 195–212. 10.1177/0143034300212006.

[CR79] U.S. Census Bureau. (2021, August 12). *Racial and Ethnic Diversity in the United States: 2010 Census and 2020 Census*. https://www.census.gov/library/visualizations/interactive/racial-and-ethnic-diversity-in-the-united-states-2010-and-2020-census.html.

[CR80] Umaña-Taylor, A. J. (2016). Ethnic-racial identity: Conceptualization, development, and youth adjustment. In L. Balter & C. S. Tamis-LeMonda (Eds.), *Child psychology: A handbook of contemporary issues* (3rd ed., pp. 305–328). Taylor & Francis.

[CR81] Umaña-Taylor, A. J. (2018). Intervening in cultural development: the case of ethnic–racial identity. *Development and Psychopathology*, *30*(5), 1907–1922. 10.1017/S0954579418000974. 30139400 10.1017/S0954579418000974

[CR82] Umaña-Taylor, A. J. (2023). Promoting adolescent adjustment by intervening in ethnic-racial identity development: opportunities for developmental prevention science and considerations for a global theory of change. *International Journal of Behavioral Development*, *47*(4), 352–365. 10.1177/01650254231162614.

[CR83] Umaña-Taylor, A. J. (in press). Leveraging ethnic-racial identity development in schools to promote ethnic-racial equity. In T. Yip (Ed.), *The Cambridge Handbook of Ethnic/Racial Discrimination and Youth*. Cambridge University Press.

[CR84] Umaña-Taylor, A. J., Kornienko, O., Douglass Bayless, S., & Updegraff, K. A. (2018). A universal intervention program increases ethnic-racial identity exploration and resolution to predict adolescent psychosocial functioning one year later. *Journal of Youth and Adolescence*, *47*, 1–15. 10.1007/s10964-017-0766-5. 29030792 10.1007/s10964-017-0766-5

[CR85] Vang, T. M., & Nishina, A. (2022). Fostering school belonging and students’ well‐being through a positive school interethnic climate in diverse high schools. *Journal of School Health*, *92*(4), 387–395. 10.1111/josh.13141. 35067912 10.1111/josh.13141

[CR86] Verkuyten, M., & Thijs, J. (2013). Multicultural education and inter-ethnic attitudes: an intergroup perspective. *European Psychologist*, *18*(3), 179–190. 10.1027/1016-9040/a000152.

[CR87] Vermunt, J. K. (2017). Latent class modeling with covariates: two improved three-step approaches. *Political Analysis*, *18*(4), 450–469. 10.1093/pan/mpq025.

[CR88] Vuong, Q. (1989). Likelihood ratio tests for model selection and non-nested hypotheses. *Econometrica*, *57*(2), 307–333. 10.2307/1912557.

[CR89] Wang, M.-T., & Eccles, J. S. (2012). Social support matters: longitudinal effects of social support on three dimensions of school engagement from middle to high school. *Child Development*, *83*(3), 877–895. 10.1111/j.1467-8624.2012.01745.x. 22506836 10.1111/j.1467-8624.2012.01745.x

[CR90] Wantchekon, K. A., & Umaña-Taylor, A. J. (2024). Targeting ethnic-racial identity development and academic engagement in tandem through curriculum. *Journal of School Psychology*, *103*, 101292 10.1016/j.jsp.2024.101292. 38432735 10.1016/j.jsp.2024.101292

[CR91] Yosso, T. J. (2005). Whose culture has capital? A critical race theory discussion of community cultural wealth. *Race Ethnicity and Education*, *8*(1), 69–91. 10.1080/1361332052000341006.

[CR92] Zimet, G. D., Dahlem, N. W., Zimet, S. G., & Farley, G. K. (1988). The multidimensional scale of perceived social support. *Journal of Personality Assessment*, *52*(1), 30–41. 10.1207/s15327752jpa5201_2. 10.1080/00223891.1990.9674095 2280326

